# A Model-Based Bayesian Estimation of the Rate of Evolution of VNTR Loci in *Mycobacterium tuberculosis*

**DOI:** 10.1371/journal.pcbi.1002573

**Published:** 2012-06-28

**Authors:** R. Zachariah Aandahl, Josephine F. Reyes, Scott A. Sisson, Mark M. Tanaka

**Affiliations:** 1 School of Mathematics and Statistics, University of New South Wales, Sydney, New South Wales, Australia; 2 Evolution & Ecology Research Centre and School of Biotechnology & Biomolecular Sciences, University of New South Wales, Sydney, New South Wales, Australia; 3 The Kirby Institute, University of New South Wales, Sydney, New South Wales, Australia; University of California San Diego, United States of America

## Abstract

Variable numbers of tandem repeats (VNTR) typing is widely used for studying the bacterial cause of tuberculosis. Knowledge of the rate of mutation of VNTR loci facilitates the study of the evolution and epidemiology of *Mycobacterium tuberculosis*. Previous studies have applied population genetic models to estimate the mutation rate, leading to estimates varying widely from around 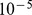 to 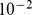 per locus per year. Resolving this issue using more detailed models and statistical methods would lead to improved inference in the molecular epidemiology of tuberculosis. Here, we use a model-based approach that incorporates two alternative forms of a stepwise mutation process for VNTR evolution within an epidemiological model of disease transmission. Using this model in a Bayesian framework we estimate the mutation rate of VNTR in *M. tuberculosis* from four published data sets of VNTR profiles from Albania, Iran, Morocco and Venezuela. In the first variant, the mutation rate increases linearly with respect to repeat numbers (linear model); in the second, the mutation rate is constant across repeat numbers (constant model). We find that under the constant model, the mean mutation rate per locus is 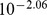 (95% CI: 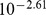,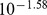)and under the linear model, the mean mutation rate per locus per repeat unit is 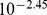 (95% CI: 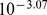,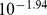). These new estimates represent a high rate of mutation at VNTR loci compared to previous estimates. To compare the two models we use posterior predictive checks to ascertain which of the two models is better able to reproduce the observed data. From this procedure we find that the linear model performs better than the constant model. The general framework we use allows the possibility of extending the analysis to more complex models in the future.

## Author Summary

Genetically typing the bacterium responsible for tuberculosis is useful for understanding the evolutionary and epidemiological characteristics of the disease. Typing methods based on variable number tandem repeat (VNTR) loci are increasingly being used. These loci, which are composed of repeated units, mutate by increasing or decreasing in the number of these repeats. Knowledge of the mutation rate of molecular markers facilitates the epidemiological interpretation of the observed genetic variation in a sample of bacterial isolates. Few studies have examined the rate of mutation at these markers and estimates to date have varied considerably. To address this problem we develop a stochastic model of evolution of these markers and then estimate their mutation rate using approximate Bayesian computation. We examine two alternative forms of the mutation process. The observed data are from four published data sets of tuberculosis bacterial isolates sampled in Albania, Iran, Morocco and Venezuela. We find that these markers have fairly high rates of mutation compared with estimates from previous studies.

## Introduction

*Mycobacterium tuberculosis*, the bacterial pathogen that causes tuberculosis, latently infects one third of the world's population and is responsible for the highest mortality rate of any single bacterial pathogen [1]. Recent advances in genotyping techniques have increased our ability to discriminate among *M. tuberculosis* isolates, helping to shed light on the genetic diversity, demographics and evolution of this pathogen [2], [3]. For instance, Pepperell et al. [4], [5] suggested that the restricted diversity in this bacterial species is likely the result of population bottlenecks and founder effects. Genotyping or fingerprinting also refines our understanding of the epidemiological characteristics of the disease in a population, for example by revealing the extent of local transmission and factors associated with this transmission (e.g., [6]).

Frequently used methods for genetic fingerprinting of *M. tuberculosis* include restriction fragment length polymorphism typing based on mobility of the insertion sequence IS *6110* [7] and spoligotyping which exploits variation at the Direct Repeat or CRISPR locus [8]. More recently, a multilocus typing method based on variable numbers of tandem repeats (VNTR) has been developed for *M. tuberculosis* [9]–[11]. These loci are minisatellites, and are also known as mycobacterial interspersed repetitive units (MIRUs). We will refer to these as “VNTR loci”.

VNTR-based methods are increasing in importance and efforts are being made to standardise the loci used [9]. The larger the number of loci used, the greater the discrimination among isolates resulting in a large number of smaller clusters of identical profiles in a sample. The early standard of 5 locus VNTR typing lacked the discriminatory power of IS*6110*-typing but comparative studies have shown that using at least 12 loci can have comparable or better discrimination relative to IS*6110* [12]–[14]. An advantage of using VNTR is that if the mutation rate is low there is the possibility of adding more loci to increase discriminatory power [10].

Inferences about transmission are sensitive to the degree of genetic clustering, which is a function of the mutation rate of the marker [15]. It is therefore important to have accurate estimates of the mutation rate of VNTR loci. Knowledge of the mutation rate of VNTR also allows calibration of the molecular clock to make inferences about the evolutionary history of *M. tuberculosis*, for instance, the time until the most recent common ancestor of a clade [3].

A standard model for the evolution of VNTR loci is the stepwise mutation model [16], [17], which has successfully been used to describe microsatellite evolution in eukaryotes (e.g. [18]). The stepwise mutation model has also been applied to VNTR evolution in *M. tuberculosis* [19], leading to estimates of the rate of mutation. Such estimates in the literature vary widely from 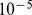 per locus per year [19] to 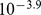 per locus per year [3] to 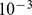
–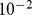 [20]. This wide variation in estimates has led to debate in the literature [21]–[24]. Taking a model-based approach can help to resolve this question. It allows our understanding of biological mechanisms underlying VNTR evolution to be incorporated into the analysis, while providing a natural framework for model validation and criticism. Similarly, examination of multiple data sets under the same models and methods could provide support or otherwise for resulting estimates.

In this study we estimate the mutation rate of VNTR markers by developing a stochastic stepwise mutation process of the evolution of genotypes through gains and losses of repeat numbers [16], [19] embedded in a model of disease transmission [25]. We consider and evaluate two alternative formulations of the stepwise mutation model under a Bayesian statistical framework, applying our methods to four geographically distinct data sets. Our study provides a posterior estimate of the VNTR mutation rate under an explicit model of evolution placed within an epidemiological context.

## Methods

### Model of the dynamics of infection and mutation of VNTR loci

In the model of disease transmission we use, 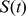 tracks the number of individuals who are susceptible to infection and 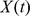 tracks infectious individuals, where 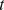 is time measured in years. For simplicity, we assume a population of fixed size 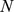. Let 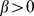 be the rate of transmission and 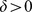 be the rate of death or recovery. First consider a deterministic model where the dynamics are given by
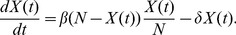
(1)We start the process with a single infected individual (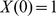). Define 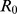 to be the basic reproductive ratio, that is, the number of cases resulting from a single infectious case in a wholly susceptible population. For this model, 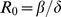. The analytical solution of Equation (1) can be written as
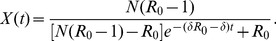
(2)The steady state of the infectious population is
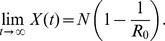



We use this deterministic model as the basis for a continuous-time stochastic model that incorporates mutation at VNTR loci. The transition rates of this model, summarised in Table 1, are as follows: the rate of new infections is 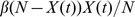 and the rate out of the infectious class from death or recovery is 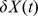. An infection event increases 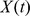 by 1 while a death-or-recovery event decreases 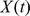 by 1. Each infection is associated with a bacterial *genotype* by which we mean the set of repeat states across all loci considered in a VNTR typing technique, determined for a particular isolate. Let 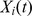 be the number of individuals infected with bacterial genotype 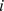 so that
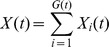
where 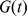 is the number of distinct genotypes in the population at time 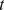.

**Table 1:** Transition rates in the stochastic model.

Event	Transition	Rate
Infection	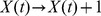	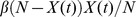
	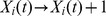	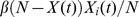
Death	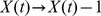	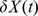
	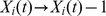	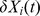
Mutation	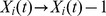	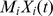
	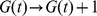 *	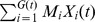
	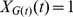 *	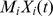

*: If an existing genotype is re-created by mutation, the count of that genotype is incremented instead. Note that the increment 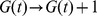 occurs before the assignment 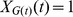.

We apply the stepwise mutation model to describe VNTR mutation [16], [17], [19] in which an event results in a unit increase or decrease in the number of repeats at a locus. We define 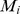 to be the mutation rate per infectious case for genotype 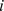 so that the transition rate for mutation of genotype 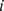 is 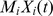. A mutation event results in either a new genotype, or a pre-existing genotype in the population (i.e., homoplasy). In the event of mutation to a new genotype, the number of individuals from the mutating genotype decreases by 1 and the number of individuals in the new class becomes 1. In the case of homoplasy, the number of individuals in the mutating genotype decreases by 1 while the number of individuals in the existing class increases by 1. In either case the total number of infected cases, 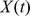, does not change.

We consider two alternative ways to specify VNTR mutation. In the first model, the mutation rate at a locus is proportional to the number of repeats at that locus. In this *linear model*, the per-locus mutation rate increases linearly with the number of repeats at the locus. In the second *constant model*, the mutation rate the per-locus mutation rate is constant and thus not dependent on repeat number. Defining 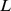 to be the number of loci, 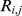 to be the number of repeats at locus 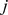 for genotype 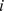, and 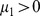 to be the rate of mutation at a locus with a single repeat, under the linear model
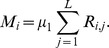
Under the constant model
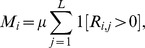
where 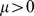 is the per locus mutation rate and where the indicator function 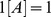 if 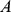 is true and 0 otherwise. In both models the boundary condition 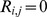 is an absorbing state in that a locus with zero repeats cannot gain or lose repeats.

The process starts at time 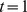 with a single infected individual and the population evolves until time 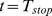. The initial individual has genotype given by 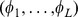, which we call the founding genotype. At time 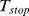 a sample of size 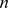 is taken from the population. We simulate this process using the Gillespie exact algorithm [26] so that the time between events is distributed exponentially, with parameter 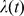, where
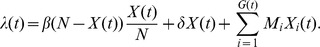
Given an event, the probability of a specific outcome is proportional to the rate of that outcome, so that
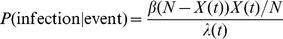


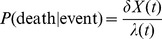


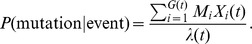
Given a mutation event, the probability of mutation in an individual with genotype 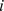 is
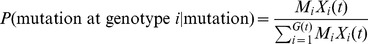
and given a mutation event in genotype 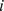, the probability that it occurs at locus 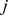 under the linear model is
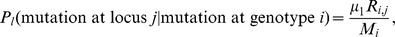
and under the constant model is
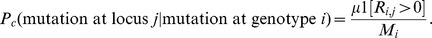
We assume that given a mutation event at locus 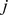 in genotype 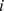, the probability of repeat gain is equal to the probability of repeat loss, following [3], [19].

### Inference procedure

We implement a standard Bayesian analysis of model parameters using approximate Bayesian computation (ABC) [27]–[29]. ABC methods permit approximate Bayesian inference when numerical evaluation of the posterior distribution is either computationally prohibitive or not available, and have been successfully applied to problems in molecular epidemiology [30]–[34].

Intuitively, given a candidate parameter vector, 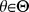, prior distribution 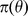 and model likelihood 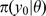 with observed data 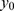, ABC methods proceed by generating an artificial dataset from the model 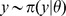 and then reducing the dataset to a low dimensional vector of summary statistics, 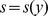. If 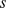 is similar to the same vector of statistics obtained from the observed data, 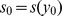, then 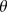 could have credibly reproduced the observed data under the model. As such, the parameter vector is then retained as part of the approximate posterior, otherwise it is discarded. More precisely, the posterior obtained under ABC methods is given by
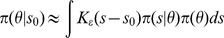
(3)where 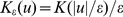 is a standard smoothing kernel with scale parameter 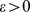. As 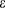 becomes small, the approximation (3) becomes increasingly accurate, although computational overheads increase. If the vector of summary statistics are informative for the model parameters, then this posterior distribution approximates the true posterior distribution so that 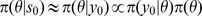. See e.g. [30], [31], [35], [36] for further description of ABC methods.

The parameter vector for the constant model above is 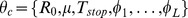 where 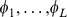 is the repeat structure of the founding genotype in the simulation. For the linear model we have 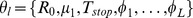. Except where this may cause confusion, we will refer to a non-model-specific parameter vector as 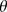.

Conditional on the parameter vector 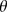, and following simulation under the model, a sample of size 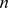 individuals is drawn from the resulting population. Summary statistics, 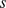, are then computed, determined as quantities expected to be highly informative regarding the model parameters. Using lower case letters (e.g. 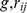) to denote sample-based values of the population-level counterparts (e.g. 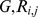), the summary statistics include the number of distinct genotypes in the sample, 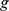, and the set of 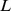 sample means of repeats at each locus
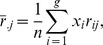
for 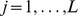, which is expected to contain information about the initial repeat numbers 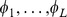 for some time after the founding case. Here, 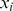 denotes the number of individuals in the sample with genotype 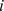, and 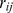 denotes the within-sample number of repeats at locus 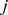 for genotype 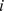. The final two statistics are based on the ANOVA decomposition 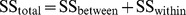 given by
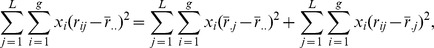
where 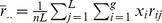, from which 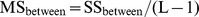 and 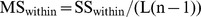 can be computed. These two statistics are expected to be informative about the mutation rate between and within loci. The complete vector of summary statistics is then given by
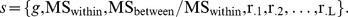
To complete the model specification, we set the parameter 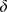 to 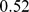, following [32], [37]. This death/recovery rate is the sum of the death rate due to tuberculosis, the death rate due to other causes, and the recovery rate from tuberculosis. We chose an informative prior distribution for 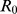 based on the study of the basic reproductive value of tuberculosis by Blower et al. [38]. We use a distribution approximating the histogram in Figure 3a in reference [38] which has a mean of 5.16 and a standard deviation of 2.82, and in particular define the prior of 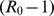 to be a gamma distribution with a shape parameter of 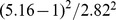 and a scale parameter of 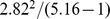. The priors for 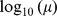, 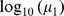, 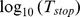 and 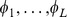 are uniform with wide ranges as shown in Table 2.

**Table 2:** Prior distributions and initial sampling distributions for each model parameter.

Parameter	Prior distribution	Initial sampling distribution
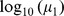	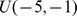	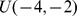
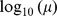	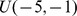	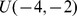
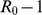	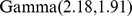	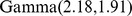
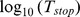	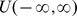	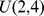
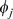	Uniform on 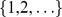	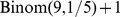

Initial sampling distributions are utilised in the ABC simulations (see Text S1).

We examine the effectiveness of the ABC inference procedure by evaluating its ability to recover accurate estimates of the mutation rate based on data generated under the constant and linear models We simulated a population of 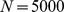 individuals with 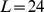 loci, 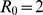, 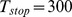, and considered a range of mutation rates under each model varying across orders of magnitude 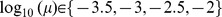 and 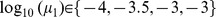. The number of repeats of the founding genotype were initialised as 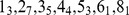 (determined as random draws from 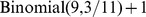), where 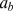 denotes 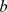 loci with repeat number 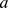. Based on a sample of size 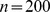 we generated data under each mutation rate value, and obtained weighted samples from the ABC posterior approximations 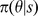 (c.f. 3) using a population-based ABC algorithm, following [32], [39], [40]. The technical algorithmic details are given in Text S1.

**Figure 1:**
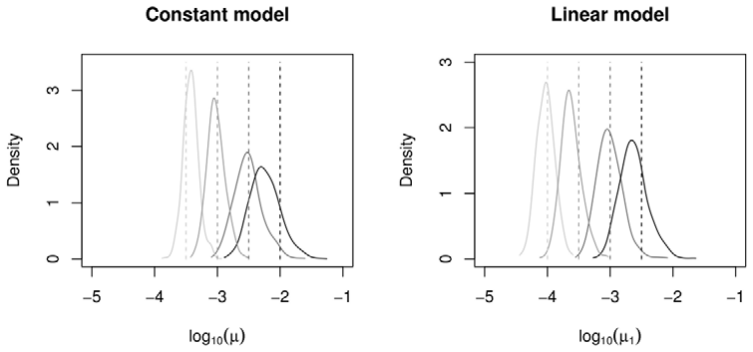
Marginal posterior distributions for 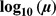 and 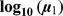 using simulated data. Plots show the marginal posterior distribution of 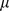 (left) and 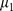 (right) using four simulated data sets generated from the constant (left) and linear (right) VNTR models. The known values of 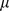 and 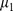 used to generate the data, 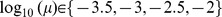 and 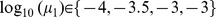, are indicated by vertical lines.

The estimated posterior distributions of 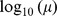 and 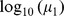 using the simulated data are shown in Figure 1. These results indicate that mutation rates can generally be recovered accurately, with the true parameter values lying in regions of high posterior density close to the posterior mode, and with a clear location shift in the density with varying mutation rate. Higher precision can be attained by using a larger sample size, although 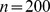 already represents a sample larger than the real datasets used for this study (c.f. Table 3). In the ABC setting, posterior precision can also be improved by reducing the kernel scale parameter 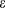 in (3) or by the inclusion of more summary statistics [30], [31], [35], [36], although each of these can substantially increase computational overheads. Improving the precision of posterior parameter estimates for given summary statistics is currently an area of active ABC research [41].

**Table 3:** Summary of data sets analysed in this study.

Country	TB incidence*	Loci	Isolates	Collection period	Source
Albania	15	24	100	2006–2007	[42]
Iran	19	15	154	2004–2005	[43]
Morocco	92	12	153	1997–1998	[11]
Venezuela	33	24	67	1997–2007	[44]

*: per 100,000 per year. Data from [57].

### Data

We selected recently published VNTR loci data sets from studies undertaken in four countries: Albania [42], Iran [43], Morocco [11] and Venezuela [44]. We chose data sets with a high number of isolates largely from the same clade, a high number of VNTR loci in the typing method, and relatively short periods of isolate collection. The data from Albania and Venezuela are based on 24-locus typing, and the data from Iran and Morocco are based on 15 and 12 loci respectively. A summary of these data are provided in Table 3, along with the incidence of tuberculosis for each country.

**Figure 2:**
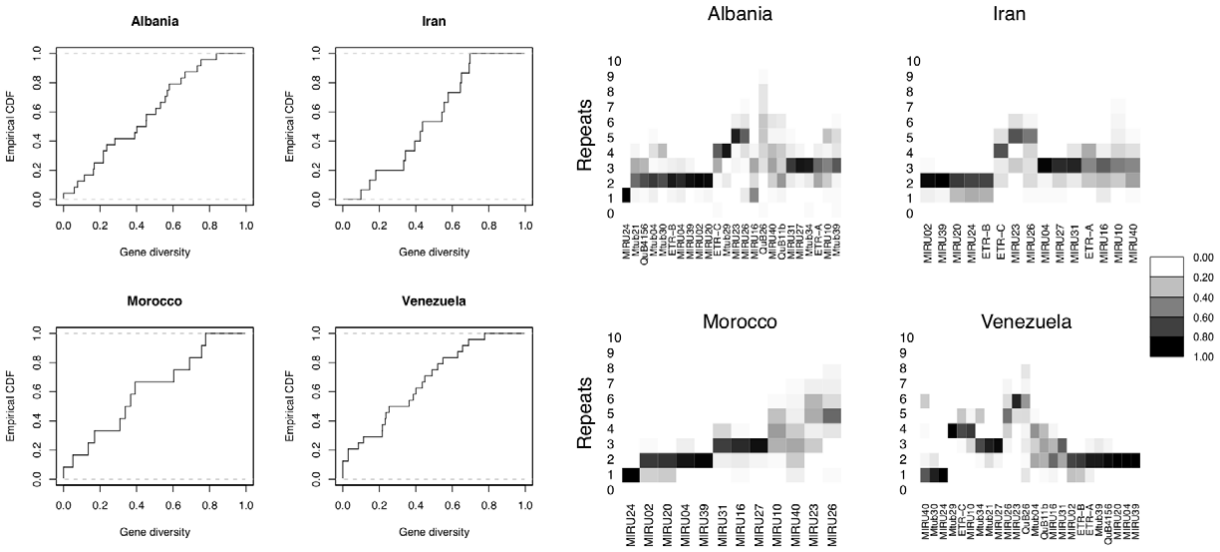
Genetic diversity of VNTR loci for each published dataset. Left plots: Empirical cumulative distribution function of gene diversity across loci. The gene diversity is computed at each locus as 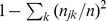 where 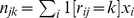 is the number of isolates with repeat size 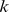 at locus 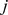. Right plots: Heat-map diversity, following Aminian et al (2009), illustrating the proportion of tandem repeats for each locus (ordered according to the original study).

As an initial exploratory examination of these data, we computed gene diversity [45] (also known as virtual heterozygosity), for each locus in each data set. This statistic is given by 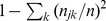 where 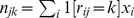 is the number of isolates with repeat size 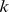 at locus 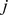. Figure 2 (left plots) shows the empirical cumulative distribution function of gene diversity across loci for each of the data sets. There is no obvious bimodality in these distributions. This feature is consistent with a common process generating diversity, compared to, for example, the potential bi- or multi-modality in the empirical cumulative distribution function arising from a multi-modal distribution of mutation rates. Similarly, plotting the proportion of VNTR states per locus per repeat (right plots of Figure 2) reveals that while some loci are more variable than others, there is no obvious separation between loci exhibiting high and low variation.

## Results

**Figure 3:**
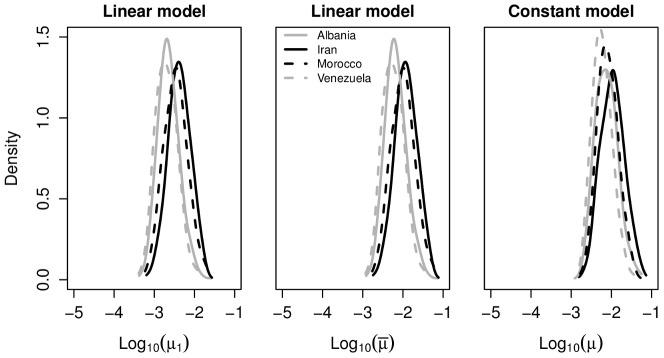
Marginal posterior estimates for 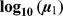, 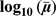 and 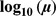. Here 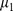 is the per-locus mutation rate for a locus with a single repeat under the linear model; 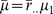 is the same quantity scaled by the mean number of repeats observed in the sample; 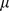 is the per-locus mutation rate for any repeat number under the constant model.

Figure 3 shows the marginal posterior distribution of the mutation rate of VNTR loci for each of the four data sets analysed. In the case of the linear model we also show (middle panel of Figure 3) the posterior of 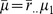, the per-locus mutation rate 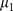 at repeat size 1 scaled by the average repeat number 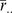 of each dataset to provide estimates of the mean per-locus mutation rate in a population with the same distribution of repeats as found in each sample. The posterior means of the mutation rate under the two models, along with 95% central credibility intervals are given in Table 4. The mean per-locus mutation rate at a locus with a single repeat from the four data sets under the linear model is 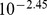, and under the constant model the mean per-locus rate is 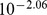. Note that the prior distributions of the mutation parameters are uniform on a logarithmic (base 10) scale, and so Figure 3 displays the posterior distributions on this scale.

**Table 4:** Bayesian posterior estimates for mutation rate.

	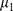		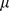	
Country	mean	95% credible interval	mean	95% credible interval
Albania	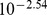	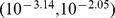	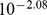	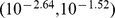
Iran	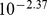	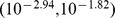	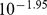	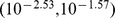
Morocco	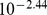	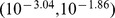	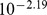	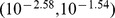
Venezuela	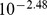	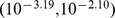	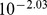	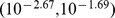

To evaluate the suitability of the constant and linear models to describe the observed data, we follow [36], [46], [47] and implement posterior predictive model checks. This approach examines the predictive distribution of specified validation statistics (based on data-generation under the fitted models) expected to be informative about various model aspects. Comparing the predictive distribution of these statistics with the same statistics derived from the observed data, enables some degree of discrimination between models. To avoid confusing model fitting with model assessment, these statistics should be different from those used in the ABC model fitting process.

Unlike the constant model, the mutation rate increases with repeat number under the linear model, and so we expect variation in repeat numbers to increase with repeat numbers. Our model assessment statistics aim to capture these differences from the data. Specifically, we focus on measures of the spread of repeats over the loci. Defining
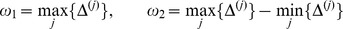
where 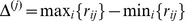, and
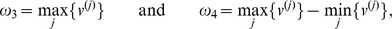
where 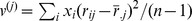, and 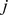 indexes loci as before, we consider the maximum (over loci) range (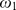), the difference between maximum and minimum range (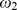), maximum variance (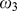) and the difference between maximum and minimum variance (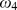).

Under the linear model, the distributions of these statistics are expected to be shifted to higher values compared to the constant model. We also fit a simple linear regression to each data set with the standard deviation of repeat number at a locus as the response variable and the mean repeat number at a locus as the predictor variable. Based on this fit, we consider
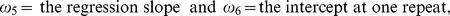
where 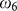 is the fitted standard deviation in repeats at a locus with a mean repeat number of one. These statistics are expected to be informative in that the slope should be positive under the linear model and near zero under the constant value, and the intercept should be low under the linear model and high under the constant model.

**Figure 4:**
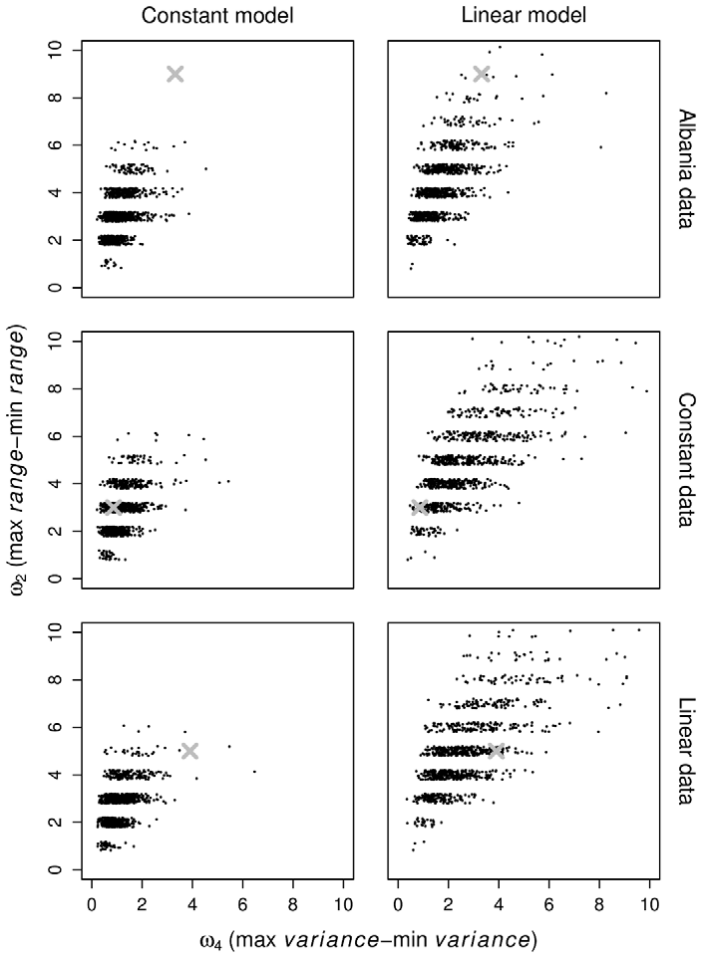
Posterior predictive model checks. Scatterplots of the posterior predictive distributions of 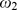 (the difference between maximum and minimum range of repeat numbers over loci), versus 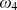 (the same quantity substituting variance for range). Columns represent constant (left) and linear (right) models. Rows represent the Albanian dataset (top), artificially generated data from the constant model (middle) and artificially generated data from the linear model (bottom). The 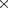 indicates the statistics derived from the observed dataset.

Figure 4 displays the predictive distributions of 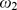 versus 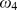 under both models. The observed data statistics are indicated by a cross (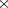). If the cross does not lie within the body of the predictive distribution, this suggests that the model and data are inconsistent with respect to aspects of the data captured by these statistics. The lower four panels present these diagnostics for artificial data generated under both models. The linear data (lower images) can be seen to be inconsistent with the constant model, but consistent with the linear model. The constant data (middle images) appear to be consistent with both models. As such, these diagnostics are able to reject the constant model when the data is generated by the linear model. In terms of the actual empirical data, the top plots in Figure 4 are based on the data from Albania. Clearly, the constant model is insufficient to describe the variation in repeat numbers inherent in the data. The linear model is better able to account for the observed pattern of repeat variation, although it is still imperfect. The posterior predictive distributions using the data sets from the other three countries were very similar to those of the Albanian data set (not shown).

**Figure 5:**
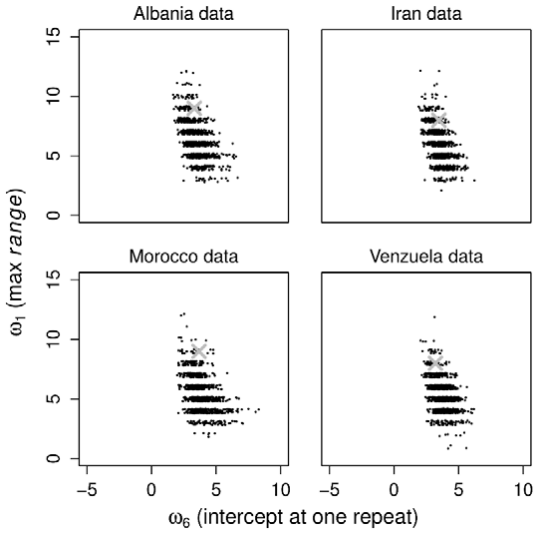
Further posterior predictive model checks. Scatterplots of the posterior predictive distributions of 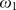 (the maximum range of repeat numbers over loci) versus 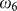 (the intercept at one repeat) under the linear model, for each observed dataset. The 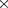 indicates the statistics derived from the observed dataset.

The question of whether the linear model is adequate is examined further in Figure 5 which shows a posterior predictive check of 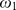 versus 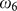 under the linear model for each of the analysed data sets. In each case, the observed data lie on the periphery of the predictive densities. Although the linear model is partially able to reproduce these statistics, this analysis shows that there is room for improvement.

## Discussion

We have analysed VNTR data from four tuberculosis studies using a model combining marker mutation and disease transmission processes, within a Bayesian framework. Our analysis shows that the VNTR mutation rate is likely to be relatively high – the posterior mean is higher than some previous estimates obtained in the literature [3], [19] and closer to more recent estimates [20]. The four data sets, which are from different geographic regions, yielded very similar estimates. Such agreement of estimates is expected if there is a common mechanism of mutation across data sets.

Previous work by two of us [20] used standard equilibrium results of the infinite alleles model to describe mutation at multiple VNTR loci, and used estimates of other markers (IS6110 and spoligotyping) to calibrate the VNTR rates. That population genetic approach did not account for evolution of VNTRs as a stepwise mutation process. It therefore did not account for homoplasy, though this problem is mitigated by the inclusion of multiple VNTR loci. Further, the underlying dynamics did not include any epidemiological details. Nevertheless, it allowed us to analyse a large number of data sets in the literature to provide a ballpark estimate of VNTR mutation rates. In contrast to that and other prior work, here we used a model that explicitly and simultaneously accounts for the mutation process of the marker and the disease dynamics, and we explored two alternative models of mutation. In addition, the stepwise mutation model used here allows mutation events to re-generate existing VNTR profiles, thereby accounting for homoplasy [48].

In the debate over the magnitude of VNTR mutation rates [3], [21]–[24] it has been noted that if loci are classified as less variable and more variable, then lower values would be estimated from the former category of loci. This raises the question of whether classification of loci into two categories of rates is supported by an underlying bimodal distribution whose modes correspond to low and high levels of polymorphism. In examining gene diversity, which is a measure of polymorphism, across loci in each data set (Figure 2) we did not observe any obvious break separating less and more variable loci. We have therefore pooled all loci and obtained an estimate of the rate of an arbitrary locus, rather than for a subset of slow or fast evolving loci. If hypermutable VNTR loci exist and are excluded from estimation procedures, using the remaining loci would clearly yield a lower mutation rate.

Our use of the linear model is a step towards resolving this issue. The linear relationship by which more units of a repeat are more prone to mutation naturally creates variation in rates. In fact, in assessing the ability of each of our two mutation models to describe the data, we found that the linear model performs better than the constant model (Figure 4). We note that the average mutation rate 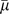 under the linear model was estimated to be very close to the mutation rate 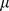 in the constant model; in this sense our analysis is robust to the exact form of the mutation model.

Despite the linear model outperforming the constant model, a posterior predictive goodness-of-fit analysis revealed some evidence that the linear model did not fit the data perfectly (Figure 5). While previous studies of eukaryote minisatellites agree with a linear relationship between repeat number and mutation rate [49], some studies of eukaryote microsatellites indicate a more complex relationship between repeat number and mutation rate [50]–[53]. We investigated a third model in which the mutation rate increases exponentially with repeat number, but the results are very similar to those of the linear model (Figure S3 in Text S1). Future work might adopt a per locus mutation rate that grows non-linearly with repeat number. A drawback of this possibility would be the added complexity and dimensionality of the model with the need to estimate further parameters in a framework that is already computationally intensive. An alternative approach might be to construct a hierarchical Bayesian model of mutation rates in which each locus is associated with its own rate according to some distribution, akin to the analysis of Bazin et al. [54].

We have used a simple model to avoid overfitting the data. However, it is possible to extend the model in future studies to incorporate further complexity and realism. One such detail is the reactivation of latent infection, which could be described by a susceptible-exposed-infected (SEI) model in which a proportion of cases progress directly to disease [38]. We performed preliminary simulations from a stochastic version of such a model (details in Text S1). We consider the number of distinct genotypes since this is one of the statistics we use in the inference and it is known to be informative for mutation rate in similar models [55], [56]. Figure S2 in Text S1 shows how the number of distinct genotypes in a sample varies with the mutation rate under both models. The latent reactivation model was able to generate statistics close to the observed statistic. The points in the region of the observed statistic are near the posterior density generated under the original model. While this is suggestive that a latency model would produce similar estimates, a full Bayesian analysis would be required to address this issue. The lack of latency is a limitation of our study which should be addressed in future research.

Migration is another factor which a more realistic multi-deme population model might incorporate. The interplay between migration and mutation may affect the resulting estimates of the mutation rate. For example, migration from regions with genetically very different clades of *M. tuberculosis* occurs at a high rate would lead to over-estimation of the mutation rate. Our approach based on the approximate Bayesian computation framework makes future directions such as this and those relating to the mutation process feasible.

## Supporting Information

Text S1 Additional technical details of the algorithm used in the Bayesian analysis, the stochastic model of latent tuberculosis reactivation, and the mutation model of VNTR with an exponential increase in rate with respect to repeat number.
(PDF)
