# Supplementary material for: A Model-Based Bayesian Estimation of the Rate of Evolution of VNTR Loci in Mycobacterium tuberculosis
Source: PLoS Comput Biol. 2012 Jun 28;8(6):e1002573. doi: 10.1371/journal.pcbi.1002573 (PMC3386166; doi:10.1371/journal.pcbi.1002573)
Supplement: Text S1 — Additional technical details of the algorithm used in the Bayesian analysis, the stochastic model of latent tuberculosis reactivation, and the mutation model of VNTR with an exponential increase in rate with respect to repeat number. (PDF) [file pcbi.1002573.s001.pdf]

Supporting Information for

A model-based Bayesian estimation of the rate of evolution of VNTR loci in *Mycobacterium tuberculosis*

R. Z. Aandahl[1], Josephine F. Reyes[2], S. A. Sisson[1], Mark M. Tanaka[2,*]
1 School of Mathematics and Statistics, University of New South Wales, Sydney, NSW, Australia
2 Evolution & Ecology Research Centre and School of Biotechnology & Biomolecular Sciences, University of New South Wales, Sydney, NSW, Australia
∗ E-mail: m.tanaka@unsw.edu.au

Bayesian algorithmic details

We sample from the approximate posterior distribution $\pi(\theta|s_0)$ (c.f. Equation 3 in the main text) using the population-based sampler of [1]. The algorithm performs a sequence of importance sampling steps $d = 1, \ldots, D$, whereby each sampling distribution is a smoothed version of the target distribution from the previous stage. Each target distribution in the sequence is defined by Equation (3) with the kernel scale parameter ϵ replaced by ϵ_d, where $\epsilon_1 > \ldots > \epsilon_D$ ensures that the approximation Equation (3) improves at each stage. The initial sampling distribution for each parameter is presented in Table 2 in the main text. For the analyses presented here, we use $N = 2000$ samples from each distribution.

The sequence $\epsilon_1, \ldots, \epsilon_D$ is dynamically constructed during algorithm implementation. The initial value ϵ_1 is set very high, to only exclude model simulations that go extinct or have zero genetic diversity. The value of ϵ_d is determined by the median of the N sampled values of $\{|s^{(d-1)} - s_0|\}$, where $s^{(d-1)}$ denotes the vector of summary statistics of an accepted parameter vector at stage $d - 1$. We use $D = 6$ importance sampling stages as computation becomes untenable beyond this. Alternative adaptive approaches for determining the sequence $\epsilon_1, \ldots, \epsilon_D$ are presented in [2, 3]

The sampling distribution at stage d is obtained by smoothing the weighted sample at stage $d - 1$ with a univariate Gaussian kernel for the parameters $R_0, \log_{10}(\mu), \log(\mu_1)$ and T_{stop}, and a binomial mass function

$$\phi_j^{(d)} - 1 \sim \text{Binomial}\left(q - 1, \frac{\phi_j^{(d-1)}}{q + 1}\right).$$

for the locus-specific parameters $\phi_1, \ldots, \phi_L$, where we set $q = 20$, which is substantially larger than the largest observed repeat number over loci (which was 10). The standard deviation of each Gaussian kernel is specified as the estimated standard deviation of the previous target distribution for that parameter, following [4].

The smoothing kernel within Equation (3), $K_\epsilon(u) = K(|u|/\epsilon)/\epsilon$, is uniform, so that $K_\epsilon(u) = \text{Uniform}(-\epsilon, \epsilon)$. To measure the distance between observed and simulated summary statistics, we use Mahalanobis distance

$$|u| = |s - s_0| = \sqrt{(s - s_0)'\Sigma^{-1}(s - s_0)},$$

where Σ is the estimated covariance matrix of $s|\tilde{\theta}$, and where $\tilde{\theta}$ is fixed in a region expected to have high posterior probability [4]. For each analysis we specified $\tilde{\theta} = \{4, 10^{-2.5}, 300, \bar{r}_{.1}, \ldots, \bar{r}_{.L}\}$. The value of $\tilde{\theta}$ was chosen through inspection of forward simulations of the model that resulted in similar levels of genetic diversity to the sample of observed isolates.

A stochastic model of latent reactivation

To explore the effect of latent infection and reactivation, we constructed a stochastic version of the model of tuberculosis dynamics proposed by [5]. The deterministic model is defined by three differential equations,

$$\begin{aligned}
\frac{dS}{dt} &= \Pi - \beta S X - \delta S \\
\frac{dL}{dt} &= (1-p)\beta S X - vL - \delta L \\
\frac{dX}{dt} &= vL + p\beta S X - (\delta + \delta_X)X,
\end{aligned}$$

where S, L, X are the densities of susceptible, latently infected and active disease classes, Π is the recruitment rate into susceptibles, β is the transmission coefficient, δ_X and δ are respectively the death rates due to tuberculosis and other causes, p is the proportion of cases entering the active disease class immediately ($1-p$ become latently infected) and v is the rate of reactivation of latent infection. We used as the initial condition a single infectious case in an otherwise susceptible population of size 50,000. A compartmental diagram of the latent reactivation model is shown in Figure S1 and the corresponding rates are described in Table S1. The model was implemented using the Gillespie exact algorithm [6]. The epidemiological parameters were set according to the values given in [5] as described in the caption of Figure S2.

Figure S2 shows how the number of distinct genotypes, g, in a sample varies with the mutation rate under both models. The implied density of the latency model simulations along the dashed line (the observed statistic for the Venezuela data) is similar to the marginal posterior distribution for $\log_{10}(\mu_1)$ obtained under the SI model, suggesting that the marginal posterior distribution under each model might be similar. Full Bayesian analysis would be needed to confirm this point.

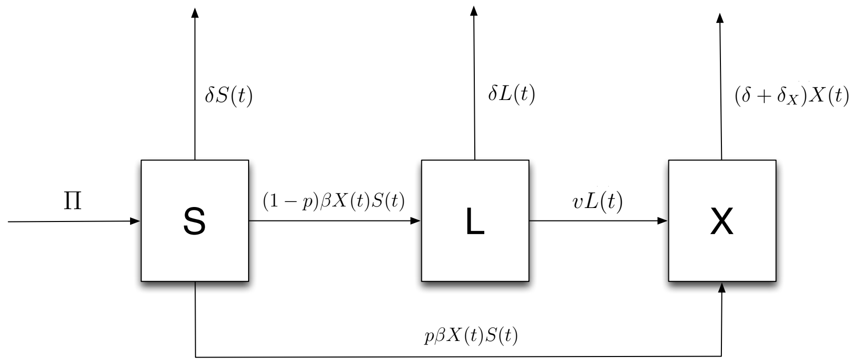

Figure S1. A compartmental diagram of the Susceptible-Exposed-Infectious model of tuberculosis transmission with latent reactivation. The arrows indicate the direction of transfer between compartments while the transition rates (more fully described in Table S1) are listed next to the arrows.

Table S1. Transition rates in the stochastic Susceptible-Exposed-Infectious model capturing latency.

Event	Transition	Rate
Birth	$S(t) \to S(t) + 1$	Π
Latent Infection	$L(t) \to L(t) + 1$	$(1 - p)\beta X(t)S(t)$
	$L_i(t) \to L_i(t) + 1$	$(1 - p)\beta X_i(t)S(t)$
Active Infection	$X(t) \to X(t) + 1$	$p\beta X(t)S(t)$
	$X_i(t) \to X_i(t) + 1$	$p\beta X_i(t)S(t)$
Latent Reactivation	$X(t) \to X(t) + 1$	$vL(t)$
	$X_i(t) \to X_i(t) + 1$	$vL_i(t)$
	$L(t) \to L(t) - 1$	$vL(t)$
	$L_i(t) \to L_i(t) - 1$	$vL_i(t)$
Susceptible Death	$S(t) \to S(t) - 1$	$\delta S(t)$
Active Death	$X(t) \to X(t) - 1$	$(\delta + \delta_X)X(t)$
	$X_i(t) \to X_i(t) - 1$	$\delta X_i(t)$
Latent Death	$L(t) \to L(t) - 1$	$\delta L(t)$
	$L_i(t) \to L_i(t) - 1$	$\delta L_i(t)$
Active Mutation	$X_i(t) \to X_i(t) - 1$	$M_i X_i(t)$
	$G(t) \to G(t) + 1$ *	$\sum_{i=1}^{G(t)} M_i X_i(t)$
	$X_{G(t)}(t) = 1$ *	$M_i X_i(t)$
Latent Mutation	$L_i(t) \to L_i(t) - 1$	$M_i L_i(t)$
	$H(t) \to H(t) + 1$ *	$\sum_{i=1}^{H(t)} M_i L_i(t)$
	$L_{H(t)}(t) = 1$ *	$M_i L_i(t)$

* If an existing genotype is recreated by mutation, the count of that genotype is incremented instead. The index i corresponds to the same genotype between the latent and active compartments. Note that the increment $G(t) \to G(t) + 1$ occurs before the assignment $X_{G(t)}(t) = 1$, and similarly for $H(t)$.

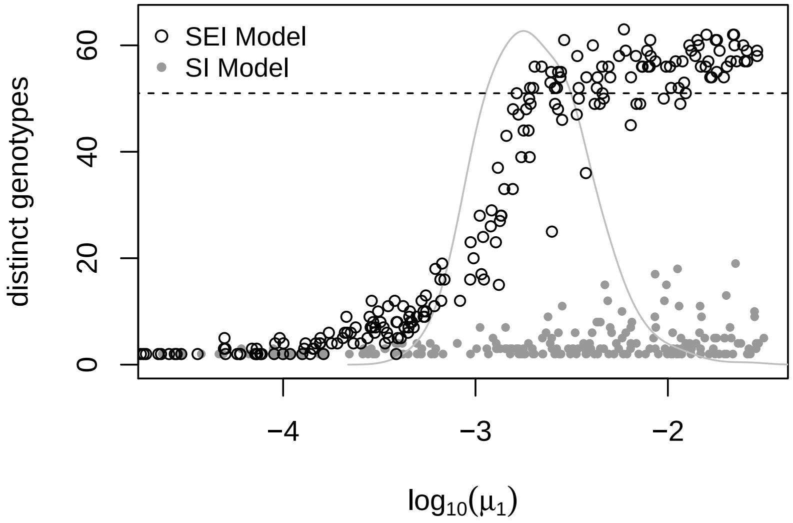

Figure S2. Prior-predictive relationship between log mutation rate and the number of distinct genotypes under alternative models. Simulations are based on the linear mutation model, with sample size and number of VNTR loci corresponding to the observed sample from the Venezuela data set. SEI Model: the Susceptible-Exposed-Infectious model for latent infection and reactivation; SI Model: the Susceptible-Infectious model without latency. The dashed line indicates the observed number of distinct genotypes from the Venezuela data set. The grey line shows the (scaled) marginal posterior distribution for $\log_{10}(\mu_1)$ for the model without latency, using the Venezuela dataset. Parameter prior distributions are those in Table 2, main text. The values of parameters other than those related to mutation are taken from Blower et al [5] as follows: $\delta = 0.02$, $\delta_X = 0.139$, $p = 0.05$, $v = 0.00392$. The values of β and Π are set as follows. Setting the disease-free equilibrium Π/δ to a total community size of 50,000, we set $\Pi = 1000$; we then derive β from $(\beta\Pi)/\delta$ which we set to 5 (similar to the value used in [5]).

An exponential model of mutation

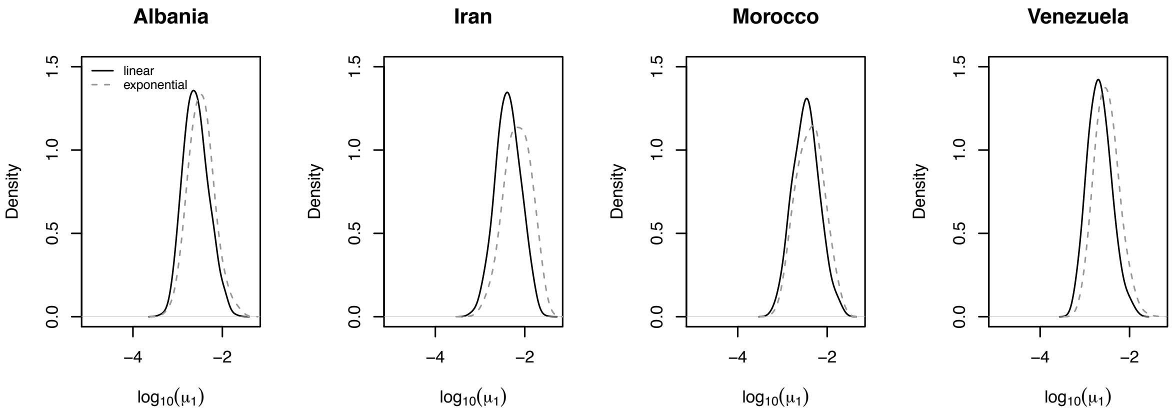

Figure S3. A comparison between marginal posterior distributions of μ_1 from the linear (solid line) and exponential (dashed line) mutation models. Here, we let the mutation rate be an exponential function of repeat number, namely, $M_i = \sum_{j=1}^{L}(e^{\mu_1 R_{i,j}} - 1)$. Note that at zero repeats, the rate is zero.

References

1. Sisson SA, Fan Y, Tanaka MM (2007) Sequential Monte Carlo without likelihoods. Proceedings of the National Academy of Sciences of the United States of America 104: 1760–1765. Errata (2009), 106, 16889.

2. Drovandi CC, Pettitt AN (2011) Estimation of parameters for macroparasite population evolution using approximate Bayesian computation. Biometrics 67: 225–233.

3. Del Moral P, Doucet A, Jasra A (2012) An adaptive sequential Monte Carlo method for approximate Bayesian computation. Statistics and Computing : in press.

4. Luciani F, Sisson SA, Jiang H, Francis AR, Tanaka MM (2009) The epidemiological fitness cost of drug resistance in *Mycobacterium tuberculosis*. Proceedings of the National Academy of Sciences of the United States of America 106: 14711–14715.

5. Blower S, Mclean A, Porco T, Small P, Hopewell P, et al. (1995) The intrinsic transmission dynamics of tuberculosis epidemics. Nature Medicine 1: 815–821.

6. Gillespie DT (1977) Exact stochastic simulation of coupled chemical reactions. The Journal of Physical Chemistry 81: 2340–2361.
